# Expansion and Delivery of Human Chondrocytes on Gelatin-Based Cell Carriers

**DOI:** 10.3390/gels11030199

**Published:** 2025-03-13

**Authors:** Krishi Patel, Derya Ozhava, Yong Mao

**Affiliations:** Laboratory for Biomaterials Research, Department of Chemistry and Chemical Biology, Rutgers University, 145 Bevier Rd., Piscataway, NJ 08854, USA; knp108@scarletmail.rutgers.edu (K.P.); do311@chem.rutgers.edu (D.O.)

**Keywords:** chondrocytes, gelatin microspheres, GelMA, microparticles, MACI, dedifferentiation

## Abstract

Cartilage damage is common in sports injuries and cartilage-related diseases, such as degenerative joint and rheumatic disorders. Autologous chondrocyte implantation (ACI) is a widely used cell-based therapy for repairing cartilage damage in clinical practice. In this procedure, a patient’s chondrocytes are isolated, cultured in vitro to expand the cell population, and then implanted into the damaged site. However, in vitro expansion of chondrocytes on standard 2D culture surfaces leads to dedifferentiation (loss of the chondrocyte phenotype), and the delivery of detached cells has proven to be ineffective. To overcome these limitations, the matrix-assisted ACI (MACI) procedure was developed. In MACI, matrices such as hydrogels and microspheres are used as cell carriers or scaffolds to deliver expanded chondrocytes, enhancing cell viability and precision delivery. To streamline the two key steps of MACI—cell expansion and delivery—this study aims to investigate various configurations of gelatin-based hydrogels for their potential to support both cell expansion and delivery as a single step. This study evaluated gelatin microspheres (Gel MS), micronized photo-crosslinked GelMA microparticles (GelMA MP), and bulky GelMA hydrogels containing cells (GelMA HG). Cell growth, maintenance of the chondrocyte phenotype, and cartilage extracellular matrix (ECM) production were assessed in pellet cultures for cells grown on/in these carriers, compared with cells cultured on tissue culture-treated polystyrene (TCP). Our results demonstrate that normal human knee articular chondrocytes exhibit robust growth on Gel MS and form aggregates enriched with glycosaminoglycan-rich ECM. Gel MS outperformed both GelMA MP and GelMA HG as a cell carrier by both supporting long-term cell growth with reduced dedifferentiation and precision delivery.

## 1. Introduction

Chondrocytes, differentiated from mesenchymal cells, are responsible for the integrity and wellness of healthy cartilage [1]. They are the only type of cells found in cartilage, making them an integral part of its makeup. Hyaline cartilage is the most abundant type of cartilage found in the human body. It is smooth and slippery, which allows bones to glide past each other without any friction or disturbance [2]. Articular cartilage is a type of hyaline cartilage that is found in the joints. Cartilage has a limited capacity of self-regeneration after injury or disease due to its avascular nature [3]. Several surgical methods, including osteochondral grafting (Mosaicplasty) and microfracture techniques, have been developed to address cartilage damage, with the choice of method depending on the severity of the injury. Osteochondral grafting is typically reserved for larger, more severe cartilage defects because of its invasive nature and the risk of donor site morbidity [4]. In contrast, microfracture is commonly used for smaller cartilage injuries. However, the clinical outcomes of microfracture procedures can vary and are often compromised by the formation of fibrocartilage instead of the more durable hyaline cartilage [5,6].

Autologous chondrocyte implantation (ACI), a regenerative cell therapy, has been widely adopted in clinical practice [7,8]. Systematic evaluations of clinical outcomes have indicated that, compared to microfracture, ACI can significantly improve patient outcomes at mid-term follow-ups of up to five years [9]. The ACI procedure consists of three main steps: harvesting chondrocytes from the patient via biopsy, expanding these cells through in vitro culture, and implanting the expanded chondrocytes into the repair site [10]. While in vitro culturing effectively increases chondrocyte numbers, it often results in the loss of their phenotype and function, a process known as dedifferentiation [11]. Normally, chondrocytes produce type II collagen and proteoglycans such as aggrecan [3,12,13]. Implanting dedifferentiated chondrocytes can compromise the quality of the regenerated cartilage [14,15].

To sustain the phenotype of chondrocytes during in vitro expansion, several strategies have been explored, including the addition of growth factors, co-culture with P_0_ primary chondrocytes or mesenchymal stem cells, and the use of low-adhesion culture surfaces or chondrocyte-derived extracellular matrix (ECM) as substrates [16,17,18,19,20]. Among these approaches, cell-derived ECM has emerged as a particular promising and scalable method for chondrocyte expansion while effectively preserving their phenotype.

In ACI, the third step is to inject the in vitro expanded autologous chondrocytes into the defect site and cover the site with a periosteal patch, which is harvested from the patient’s own bone. The implantation of detached chondrocytes without a proper scaffold often leads to a high likelihood of graft failure, delamination, and tissue hypertrophy [21,22]. To address this issue, the next generation of cell-based cartilage repair has been developed: matrix-assisted chondrocyte implantation (MACI). In this therapy method, chondrocytes obtained from the patient are expanded conventionally on a 2D substrate and loaded into 3D biocompatible scaffolds, most often collagen or hyaluronan-based matrices, which are then implanted on the patient’s site of injury [23]. This approach allows for higher cell delivery precision and better cell survival after implantation.

Various natural, biological, and synthetic materials have been explored for these matrices, including gelatin, collagen, glycosaminoglycans, fibrin, and polyacrylamide [24]. Porous collagen scaffolds and hydrogels are the most used matrices for culturing and delivering chondrocytes [24,25]. Collagen, a major component of native ECM, is highly biocompatible and biodegradable. However, it remains relatively expensive and can provoke immunogenic responses in in vivo studies [26]. Glycosaminoglycans, while essential components of healthy cartilage, often lack cell adhesion sites and can even inhibit chondrogenesis when used excessively in in vivo applications [27]. On the other hand, in recent years, more cost-effective and widely available biomaterials such as gelatin and methacrylated gelatin (GelMA) have demonstrated their versatility and biocompatibility in supporting the growth and delivery of many types of cells, including stem cells and chondrocytes [28,29,30,31]. Specifically, GelMA hydrogels have been shown to effectively encapsulate human chondrocytes, maintain their viability, and deliver them to superficial cartilage defects [29]. Despite their advantages, chondrocytes encapsulated in GelMA hydrogels often exhibit limited proliferation, likely due to restricted nutrient accessibility and physical constraints [32,33,34]. In one study, after four weeks of in vitro culture, no significant change in DNA content was observed within the hydrogels, indicating that while hydrogel encapsulation is effective for cell delivery, it may not be sufficient as a scaffold for supporting long-term cell growth and functionality [33].

Beyond encapsulation, hydrogels, including gelatin-based hydrogels, can also serve as carriers for cells or biomolecules in the form of microparticles [35]. Our previous studies demonstrated that cells cultured on the surface of hydrogel microparticles exhibited improved proliferation and maintained functionality more effectively compared to encapsulated cells [36]. This enhanced growth is likely attributed to the increased surface area provided by hydrogel microparticles, improved nutrient accessibility, and more efficient waste removal compared to encapsulation systems. These benefits encourage further exploration of gelatin-based microparticles as both culturing scaffolds and delivery systems for chondrocytes. By supporting chondrocyte expansion and enabling efficient delivery, gelatin-based microparticles have the potential to integrate these two processes into a single-step approach for the MACI procedure.

Gelatin microparticles have been investigated as carriers for chondrocyte delivery to support cartilage repair [37]. Leveraging gelatin’s temperature-sensitive liquid–gel transition, Leong et al. developed an innovative strategy to encapsulate porcine chondrocytes within gelatin microspheres at low temperature before embedding them in alginate hydrogels. Upon heating at 37 °C, the gelatin microspheres dissolved, releasing chondrocytes into the hydrogel and creating porous structures for cell expansion [38]. However, due to the limited bioactivity of alginate hydrogels, this approach was later refined by encapsulating chondrocytes directly within the more bioactive GelMA hydrogel [29]. Cruz et al. further investigated crosslinked stable small gelatin microspheres (~10 μm) as chondrocyte carriers. In their study, human chondrocytes were first expanded on conventional 2D tissue culture plates (TCPs), a process known to promote dedifferentiation. However, when combined with gelatin microspheres, in an aggregate culture, the dedifferentiated chondrocyte phenotype was partially reversed, particularly in the presence of TGF-β [39]. This study highlighted the biocompatibility and therapeutic potential of gelatin microspheres for cartilage repair. Beyond chondrocyte delivery, gelatin microspheres have also been widely employed as carriers for stem cells and various growth factors and cytokines to promote cartilage regeneration [37,40].

Despite the use of gelatin-based materials as scaffolding matrices to facilitate cell delivery, MACI applications of these studies still required a two-step process: expansion on TCP to achieve a sufficient yield, which induces dedifferentiation, and cell loading onto carriers for implantation. This study aimed to streamline the MACI procedure by integrating both chondrocyte expansion and delivery onto gelatin-based carriers in a single, more efficient step.

In this study, three configurations of gelatin-based hydrogels—gelatin microparticles (Gel MSs), GelMA hydrogels (GelMA HGs), and GelMA microparticles (GelMA MPs)—were prepared. Their ability to streamline the MACI procedure by supporting human chondrocyte growth and phenotype maintenance, including glycosaminoglycan production, was directly compared to standard 2D culture conditions on tissue culture-treated polystyrene (TCP). Our results indicate that Gel MSs serve as a promising scaffold for MACI procedures, effectively integrating cell expansion and delivery while minimizing differentiation. This streamlined approach eliminates the need for an extensive two-step process.

## 2. Results and Discussion

### 2.1. Preparation of Gelatin-Based Carriers

**Figure 1:**
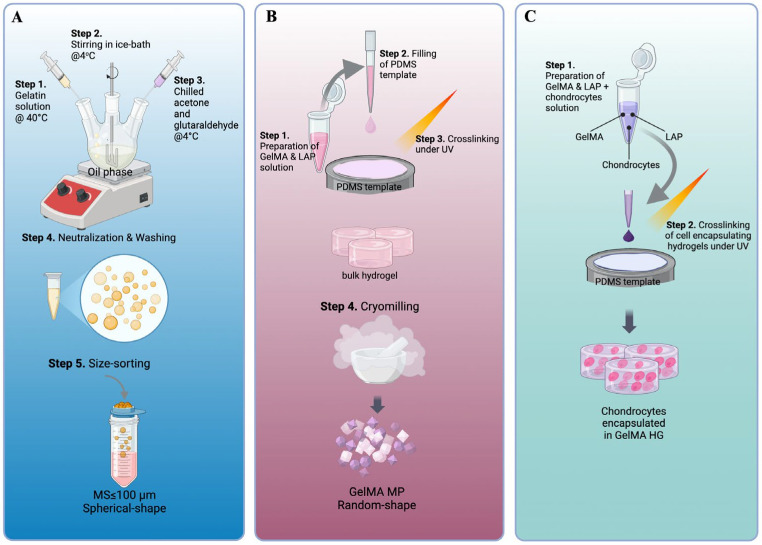
Key steps of the fabrication processes of gelatin-based cell carriers. The key steps of making gelatin microspheres (Gel MSs) (**A**), GelMA microparticles (GelMA MPs) (**B**) and GelMA hydrogels (GelMA HGs) (**C**) are illustrated.

GelMA hydrogels (GelMA HGs) have been used as cell carriers for chondrocytes [29,32,34]. To explore hydrogel microparticles as an alternative carrier configuration, two types of gelatin-based microparticles were prepared. As described in Figure 1A, gelatin microspheres (Gel MSs) were fabricated using a water-in-oil emulsion technique with gelatin as the starting material, followed by glutaraldehyde crosslinking. Previous studies have shown that the size of microspheres (MSs) significantly impacts cell growth on their surfaces. Specifically, MSs with diameters smaller than 100 µm appear to be preferred by human stem cells [36]. To meet this criterion, Gel MSs smaller than 100 µm were isolated using 100 µm cell strainers. The lyophilized Gel MSs remained stable at 4 °C for at least six months.

Unlike the chemical crosslinking used for Gel MSs, GelMA HG crosslinking was initiated with a photoinitiator and completed under 405 nm light. This mild crosslinking approach is essential to ensure cell viability during the encapsulation process (Figure 1C) [41]. To enhance the surface area available for cell interactions, GelMA HGs were cryomilled into microparticles (Figure 1B). The resulting microparticles were lyophilized for storage.

**Figure 2:**
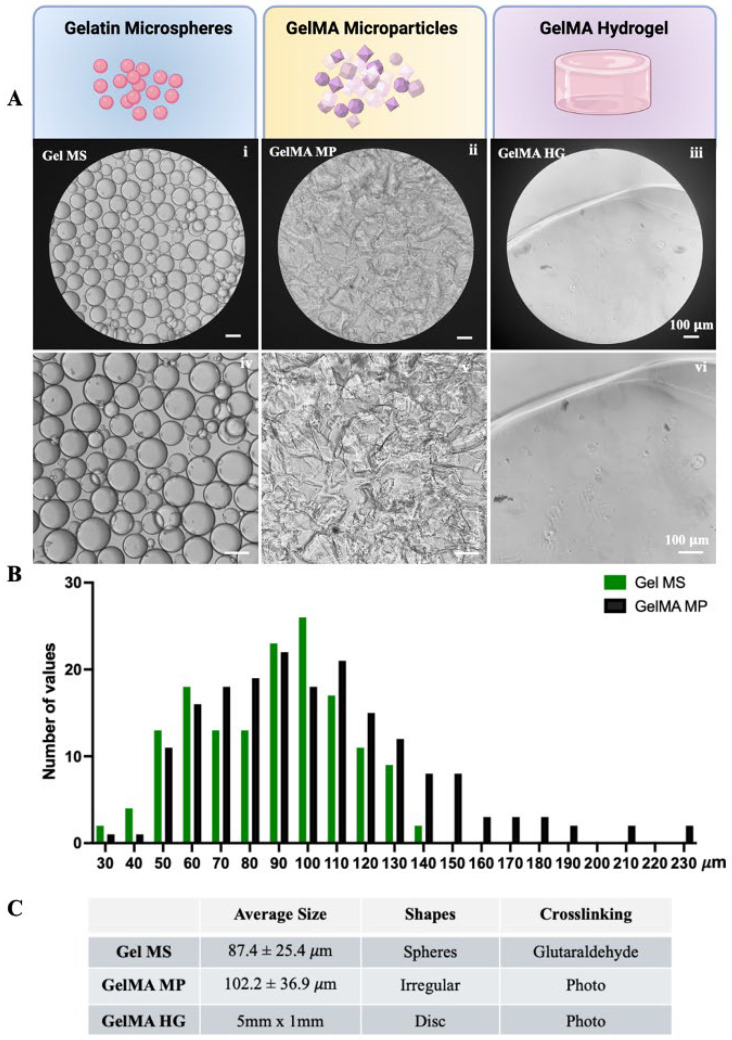
Characteristics of gelatin-based cell carriers. (**A**) The phase contrast images of rehydrated Gel MSs (**i**,**iv**), GelMA MPs (**ii**,**v**) and freshly prepared GelMA HGs (**iii**,**vi**) were captured using a light microscope. Representative images are shown. Scale bar = 100 µm. (**B**) The size distributions of Gel MS samples (green) and GelMA MP samples (black) were graphed using GraphPad Prism. (**C**) The average sizes of the Gel MSs and GelMA MPs were calculated from at least 200 measurements. Data shown are mean ± SD.

Upon rehydration, Gel MSs formed microspheres with smooth surfaces (Figure 2A(i,iv)). The diameters of the Gel MSs were measured using ECHO microscope software (Revolve-M395), based on phase-contrast images captured with an ECHO microscope (Supplementary Figure S1). The size distribution indicated that the majority of the microspheres had diameters smaller than 100 µm, with an average diameter of 87.4 ± 25.4 µm (Figure 2B,C). In contrast, GelMA MPs exhibited irregular shapes and a broader size range (Figure 2A(ii,v)). When measured along the longitudinal axis of each particle, the size distribution was wider than that of the Gel MSs, with an average length of 102.2 ± 36.9 µm (Figure 2B,C). Even though Gel MSs and GelMA MPs are derived from different gelatin-based materials, undergo distinct processes, and adopt different shapes through crosslinking, their function as cell carriers for chondrocytes was directly evaluated as individual entities.

For comparison, bulk GelMA HGs, shaped as disks (5 mm in diameter and 1 mm in thickness), had smooth surfaces and were transparent (Figure 2A(iii,vi)).

### 2.2. Culturing Chondrocytes on Cell Carriers and TCP

To compare the growth of chondrocytes on various cell carriers, 3 mg/sample of dry Gel MS or GelMA MP was rehydrated. In total, 9 × 10^4^ normal human articular chondrocytes (NHACs) were seeded onto each Gel MS or GelMA MP sample. To encapsulate the NHACs within GelMA HGs, the NHACs were mixed with a 10% GelMA solution containing photoinitiators. The resulting mixture was aliquoted into polydimethylsiloxane (PDMS) molds (5 mm in diameter and 1 mm in depth) and crosslinked using a 405 nm UV lamp. Each GelMA HG sample (disk) contained 3 mg of GelMA (dry weight) and 9 × 10^4^ NHACs (Figure 1C). As a control, 9 × 10^4^ NHACs were seeded per well onto standard 6-well tissue culture plates (TCPs).

**Figure 3:**
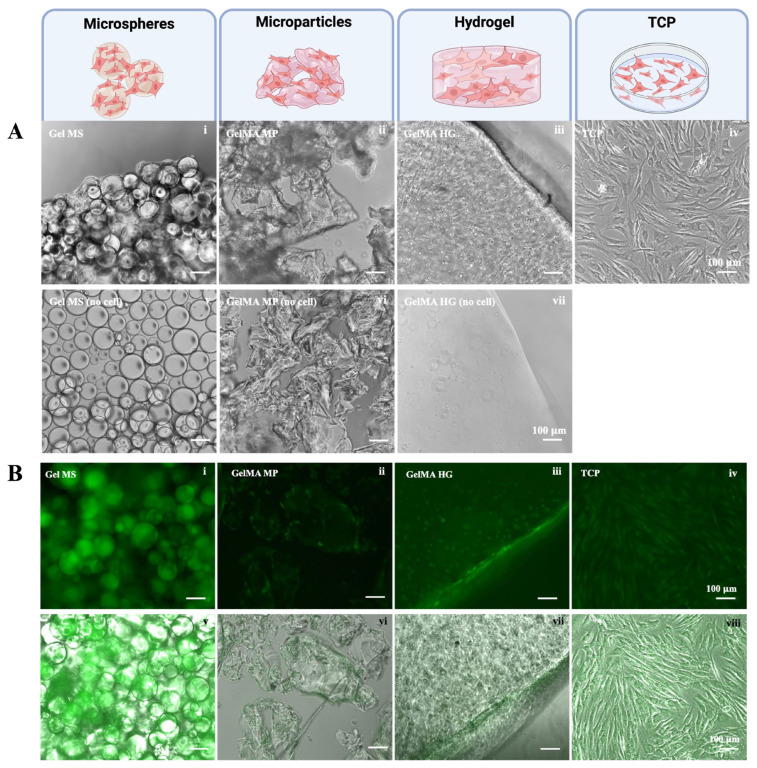
Growth of NHACs on different cell carriers. (**A**) Representative phase contrast images of NHACs cultured on Gel MSs (**i**), on GelMA MPs (**ii**), in GelMA HGs (**iii**), or on TCPs (**iv**) for four days are shown. The cell carriers in the absence of cells are shown (**v**–**vii**). Scale bar = 100 µm. (**B**) The CalceinAM staining of cells on Gel MSs (**i**), on GelMA MPs (**ii**), in GelMA HGs (**iii**), or on TCPs (**iv**) were examined under a fluorescent microscope. The fluorescent images merged with their phase contrast images (**v**–**viii**) are shown in the lower panel. Representative images are shown. Scale bar = 100 µm.

After four days of culturing the cells in growth medium (DMEM base medium containing 10% FBS and 1× antibiotic-antimycotic), the cell carriers without cells remained dispersed (Figure 3A(v–vii)). In contrast, in the presence of NHACs, the Gel MS were covered with cells and formed aggregates (Figure 3A(i)). While cells were not visually discernible on GelMA MPs, the particles became less transparent compared to GelMA MPs without cells (Figure 3A(ii)). The aggregation of GelMA MPs occurred sporadically and was less extensive than the aggregation observed with Gel MSs in the presence of cells.

On the surface of the GelMA HGs, NHACs displayed elongated morphologies with limited spreading within the hydrogel (Figure 3A(iii)). In comparison, NHACs on TCPs exhibited more extensive spreading, both longer and wider, than cells on the surfaces of GelMA HGs (Figure 3A(iv)).

To visualize viable cells on the cell carriers, cells were stained with Calcein Acetoxymethyl (CalceinAM) after being cultured for seven days. CalceinAM produces green fluorescence when converted by metabolically active (live) cells [42]. As shown in Figure 3B, strong green fluorescence was observed in cells on Gel MSs (Figure 3B(i,v)). The fluorescence intensity of cells on Gel MSs was the highest among all tested groups. Green fluorescence was primarily detected along the edges or surfaces of GelMA MPs (Figure 3B(ii,vi)). Similarly, NHACs exhibited strong metabolic activity on the surfaces of GelMA HGs (Figure 3B(iii,vii)). The viable cells within the hydrogel largely maintained a round morphology. As expected, NHACs on TCPs displayed uniform viability across the surface (Figure 3B(iv,viii)).

This observation aligns with the results reported previously [36]. The lower cell growth on GelMA HGs was possibly due to inefficient nutrient and waste exchange within the hydrogel. Cell growth primarily occurred on the surface of the hydrogel, where liquid exchange was more readily accessible. Additionally, while 405 nm treatment for crosslinking is mild, it may generate oxidative stress, potentially compromising the viability of cells embedded within the hydrogel [41,43,44].

The metabolic activity (Figure 3B) of NHACs on GelMA MPs was not significantly higher than that on GelMA HGs, which was unexpected given the larger surface area of GelMA MPs compared to GelMA HGs. Previous studies using human dental stem cells showed better viability on the GelMA MPs than GelMA HGs [45]. As surface morphology and curvatures have significant impacts on cellular behaviors, this discrepancy may be attributed to the response of different cell types to variations in surface topology and curvature between the micronized particles and bulk hydrogels [46,47,48]. Whether chondrocytes are negatively impacted by the surface topology of GelMA MPs requires further investigation.

### 2.3. Pellet Culturing of NHAC on Gelatin-Based Carriers

The pellet culture of chondrocytes is often used as an in vitro model to mimic cartilage formation or the production of cartilage-like ECM [49,50]. Chondrocytes after expansion on TCPs are trypsinized and centrifuged to form a compact pellet. The high-density cell–cell interaction stabilizes the pellet over time without dispersing packed cells. NHACs cultured on Gel MSs, on GelMA MPs, or in GelMA HGs for seven days were pelleted by centrifugation without removing the carriers. GelMA HG samples stayed intact after the pelleting process. Cells cultured on TCPs were trypsinized and pelleted by centrifugation. The pellets were then cultured with minimal disturbance for 14 days.

**Figure 4:**
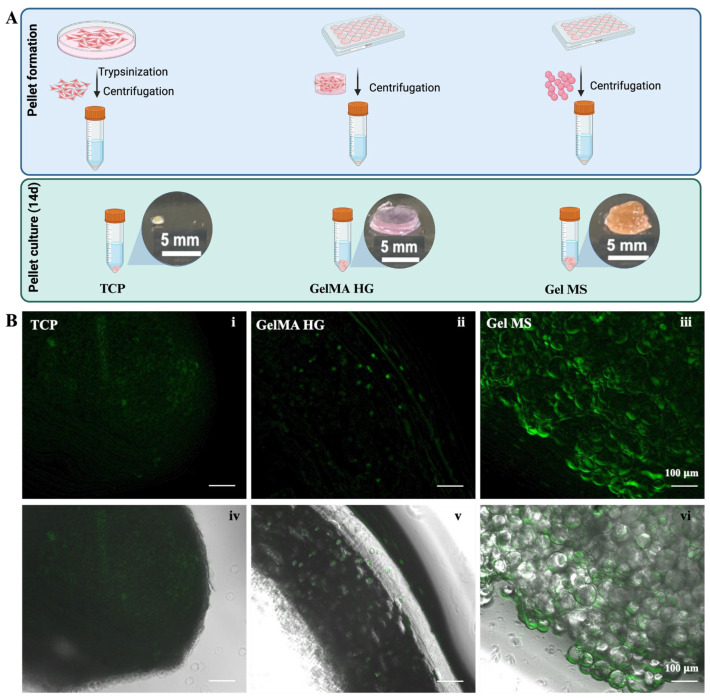
Pellet culturing of cells on carriers or from TCPs. (**A**) Illustration of pellet making and culturing. The gross observation of pellets after cultured for 14 days. (**B**) CalceinAM staining of cells in pellets of TCPs (**i**), GelMA HGs (**ii**), or Gel MSs (**iii**) were examined under fluorescent microscope. Fluorescent images merged with their phase contrast images (**iv**–**vi**) are shown in the lower panel. Representative images are shown. Scale bar = 100 µm.

Cells from TCPs, GelMA HGs, or Gel MSs remained in the form of stable pellets after 14 days (Figure 4A). However, cells on GelMA MPs failed to form stable pellets, likely due to the irregular shape of GelMA MPs and less robust cell growth. Due to its inferior ability to support chondrocyte growth and pellet formation, GelMA MPs are unlikely to be a promising cell carrier candidate, and no further analysis was conducted for cells on GelMA MPs.

The pellet formed by cells cultured on TCPs was smaller in volume compared to the aggregates formed with Gel MSs or GelMA HGs, which include the cell carriers. To visualize viable cells in the pellets, samples were stained with CalceinAM (Figure 4B). The green fluorescence in Gel MS pellets was stronger than in pellets from cells cultured on TCPs or GelMA HGs (Figure 4B(iii) vs. Figure 4B(i,ii)). Additionally, the viability distribution appeared uniform throughout the Gel MS aggregates. In contrast, fluorescence in TCP pellets was dimmer compared to cells cultured on the TCP surface (Figure 4B(i) and Figure 3B(iv)), suggesting that high-density cell packing reduces metabolic activity and proliferation, a phenomenon previously reported [17,51].

**Figure 5:**
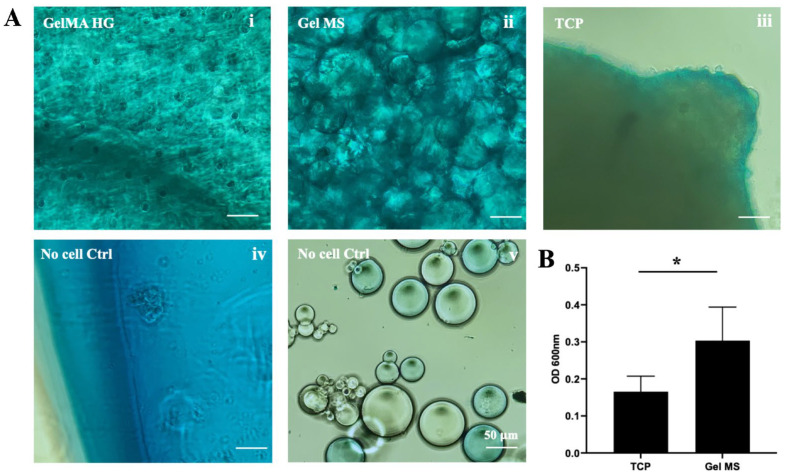
Alcian Blue staining of pellets. (**A**) Representative images of pellets from cells cultured in GelMA HGs (**i**), on Gel MSs (**ii**) or TCPs (**iii**) stained with Alcian blue dye. The cell carriers without cells were stained with Alcian Blue as background controls (**iv**,**v**). Scale bar = 100 µm. (**B**) Quantification of the Alcian Blue dye bound to pellets. The absorbance reading of cells on Gel MSs was subtracted with the reading of the carrier control. Data shown are mean ± SD. * *p* < 0.05.

Chondrocytes produce glycosaminoglycan (GAG)-enriched ECM when cultured in pellets [52,53]. To evaluate GAG production in various pellets, samples were stained with Alcian Blue dye, which specifically binds GAGs (Figure 5A). After extensive washing, pellets formed from cells cultured on TCPs showed blue staining throughout (Figure 5A(iii)). GelMA HGs with cells also showed positive staining with Alcian Blue; however, GelMA HG controls (without cells) exhibited significant background staining (Figure 5A(i,iv)). The cause of this high background staining remains unclear, but it interfered with the quantification of Alcian Blue dye for GelMA HG samples.

Pellets of Gel MSs with cells displayed strong Alcian Blue staining, with a fibrillar matrix-like structure observed, particularly between microspheres (Figure 5A(ii)). In contrast, Gel MSs without cells showed only low background staining (Figure 5A(v)). To quantify the bound Alcian Blue dye, pellets were digested with collagenase, and the released dye was quantified by absorbance at 600 nm (Figure 5B). Due to the unexplained high background in control GelMA HG samples, no positive values were obtained from stained GelMA HG-containing cells.

Even after subtracting background staining, the levels of Alcian Blue dye released from Gel MS pellets were significantly higher than those from TCP pellets. These results highlight Gel MSs as a better scaffold for supporting chondrocyte growth and the production of GAG-rich ECM.

### 2.4. Cell Expansion and Chondrocyte Phenotype Maintenance on Gel MSs, GelMA HGs, or TCPs

While MACI procedures leverage scaffolds or carriers for the precise delivery of expanded chondrocytes, many still rely on standard 2D culture methods for chondrocyte expansion [54]. If a scaffold or carrier could facilitate both expansion and delivery, it would significantly streamline the MACI procedure. Standard 2D culture is an efficient method for expanding chondrocytes through multiple passages. However, this approach is accompanied by the loss of the chondrocyte phenotype (dedifferentiation) [11]. In contrast, culturing chondrocytes on hydrogels has been shown to reduce dedifferentiation [55]. To address this, the expansion of cell numbers and the maintenance of phenotype under various culture conditions were compared.

**Figure 6:**
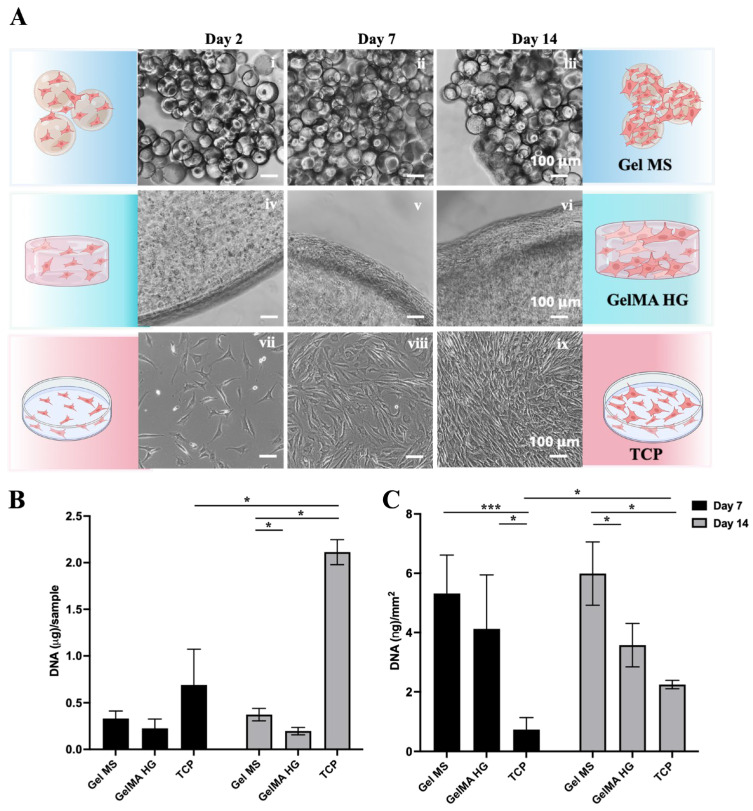
Expansion of NHACs on Gel MSs, GelMA HGs, and TCPs. (**A**) Representative phase contrast images of cells on Gel MSs (**i**–**iii**), GelMA HGs (**iv**–**vi**), or TCPs (**vii**–**ix**) for Day 2, 7, and 14. Scale bar = 100 µm. (**B**) DNA isolated from cells on Gel MSs, GelMA HGs, or TCPs on Day 7 and Day 14 was quantified using AccuClear DNA quantification kit. (**C**) The DNA from each sample was normalized to the surface areas calculated for each carrier. Data shown are mean ± SD. * *p* < 0.05, *** *p* < 0.005.

NHACs were seeded onto Gel MSs or TCPs or encapsulated in GelMA HGs and cultured for 7 and 14 days (Figure 6A). Cell proliferation was assessed by extracting and quantifying DNA from each culture at different time points (Figure 6B).

As expected, NHACs showed significant growth on TCP from Day 2 to Day 14 (Figure 6A(vii–ix)). Cells grew both around and between Gel MS, leading to the aggregation of Gel MS with cells (Figure 6A(i–iii)). In contrast, cells encapsulated in GelMA HGs remained mostly round, with spreading and growth primarily observed on the surfaces of GelMA HGs (Figure 6A(iv–vi)).

To quantify the DNA content of cells on each carrier, samples were digested with proteinase K, and DNA was extracted using phenol–chloroform–isoamyl alcohol, followed by quantification with an AccuClear dsDNA Quantification Kit (Biotium, Fremont, CA, USA) (Figure 6B). On Day 7, the DNA quantity for cells on Gel MSs was higher than on GelMA HGs. By Day 14, only minimal increases in cell density (DNA quantity) were observed on Gel MSs and GelMA HGs, whereas cells on TCPs showed a significant increase.

Since the surface areas of the three carriers differed, DNA levels were normalized to the available surface area. The total surface area of Gel MSs was estimated by determining the number of microspheres in 3 mg of Gel MSs using a hemocytometer (Hausser Scientific, Horsham, PA, USA). The total surface area of Gel MSs (~62.4 mm^2^) was calculated based on their measured diameters. The surface areas of GelMA HGs and TCPs were calculated based on their dimensions, yielding 55 mm^2^ and 940 mm^2^, respectively. After normalization, cell density was highest on Gel MSs (Figure 6C), suggesting that cells on Gel MSs either formed denser aggregates or adopted a smaller, less spread morphology.

Previous studies have reported that increased cell spreading is positively correlated with chondrocyte dedifferentiation [56]. Therefore, more compact cell growth observed on Gel MSs compared to TCPs and GelMA HGs may be advantageous for maintaining the desired cell phenotype. The lack of further increase in DNA quantity on Gel MSs or GelMA HGs after Day 7 suggests that the available surface area may have been fully occupied by that time.

**Figure 7:**
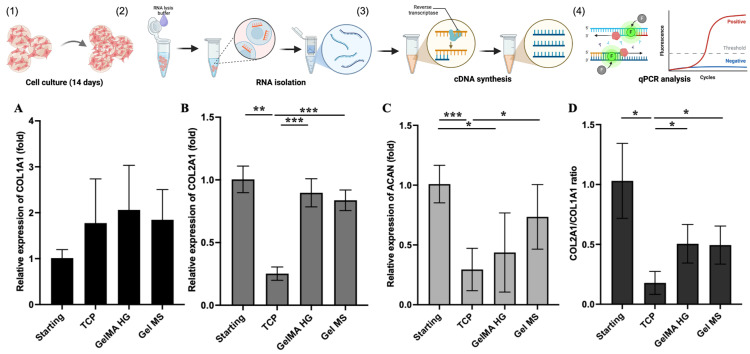
The relative gene expression of chondrocyte markers in NHACs on cell carriers or TCPs. NHACs were cultured on Gel MSs, GelMA HGs, or TCPs for 14 days (1), total RNA was isolated (2), followed by cDNA synthesis (3), and qPCR analysis (4). The relative expression of COL1A1 (**A**), COL2A1 (**B**), ACAN (**C**), or ratio of COL2A1/COL1A1 (**D**) was analyzed by quantitative PCR. Data shown are mean ± SD. * *p* < 0.05, ** *p* < 0.01, *** *p* < 0.005.

To evaluate the maintenance of the chondrocyte phenotype on different surfaces, we compared the expression of key chondrocyte marker genes in NHAC cells cultured on Gel MSs, GelMA HGs, and TCPs (Figure 7). Over 14 days of culture, the expression of type II collagen (COL2A1) and aggrecan (ACAN) genes significantly decreased in cells cultured on TCPs, compared both to the starting cells and to cells cultured on Gel MSs (Figure 7B,C). While COL2A1 expression appeared to be maintained in cells cultured in GelMA HGs, ACAN expression declined compared to the starting cells. The ratio of COL2A1 to COL1A1 expression is commonly used as an indicator of chondrocyte dedifferentiation [20]. As shown in Figure 7D, this ratio was significantly reduced in cells cultured on TCPs after 14 days, whereas cells cultured on Gel MSs or in GelMA HGs maintained comparable higher COL2A1/COL1A1 ratios.

These findings indicate that cells cultured on Gel MSs or GelMA HGs better maintained their chondrocyte phenotype compared to cells cultured on TCPs.

Consistent with previous studies, encapsulation in GelMA HGs mitigated chondrocyte dedifferentiation [55]. However, NHAC cells did not proliferate efficiently when encapsulated in GelMA HGs, indicating their inability to fulfill both critical functions: promoting cell expansion while reducing dedifferentiation. In contrast, chondrocytes cultured on Gel MSs demonstrated significant proliferation while maintaining reduced levels of dedifferentiation. These findings suggest that Gel MSs may serve as promising cell carriers to streamline the MACI procedure by integrating cell expansion and delivery into a single step.

A limitation of the current study is that Gel MSs had a smaller available surface area compared to TCPs, resulting in reduced cell expansion (total DNA quantity). This observation prompted us to explore alternative configurations of Gel MSs, such as using lower-density microspheres (by reducing the gelatin concentration during Gel MS fabrication) and incorporating cartilage ECM components.

To establish Gel MSs as a viable candidate for MACI, further characterization and evaluation are essential. The physical properties and degradation rate of Gel MSs can potentially be modulated by adjusting the gelatin concentration or altering crosslinking conditions. Optimizing the relationship between these characteristics and the performance of Gel MSs as chondrocyte carriers is critical. Moreover, chondrocytes expanded on Gel MSs must undergo evaluation in preclinical animal models to validate their functionality and therapeutic potential.

## 3. Conclusions

The prevalence of cartilage defects caused by injuries and degeneration underscores the need for effective repair strategies to alleviate pain and improve patients’ quality of life. To enhance and streamline the matrix-assisted autologous chondrocyte implantation (MACI) procedure, this study aimed to identify a gelatin-based cell carrier that supports chondrocyte expansion with reduced dedifferentiation while also serving as a delivery scaffold. Among the three hydrogel configurations tested (Gel MSs, GelMA MPs, and GelMA HGs), Gel MSs emerged as the most promising candidate. Chondrocytes cultured on Gel MSs exhibited greater proliferation, formed aggregates, and maintained their chondrocyte phenotype better than those cultured on the other carriers or tissue culture-treated polystyrene (TCP). This in vitro study identifies Gel MSs as potential cell carriers capable of integrating both cell expansion and delivery for MACI, eliminating the need for the 2D expansion of chondrocytes followed by delivery using carriers. These findings warrant further in vivo evaluation of Gel MSs in preclinical animal models to validate their therapeutic potential.

## 4. Materials and Methods

### 4.1. Making Methacrylated Gelatin Microparticles (GelMA MPs)

The synthesis and characterization of methacrylated gelatin (GelMA) was conducted as previously described [57]. To prepare GelMA MPs, 10 mL of 10% (*w*/*v*) solution of GelMA was prepared by dissolving 1 g of GelMA powder in 10 mL dH2O at 37 °C. Photoinitiator lithium phenyl-2,4,6-trimethylbenzoylphosphinate (LAP, Sigma Aldrich, St. Louis, MO, USA) at a final working concentration of 0.05% (*w*/*v*) was then added to the GelMA solution and mixed. The mixture was transferred to wells of a polydimethylsiloxane (PDMA)-coated 6-well plate (1.25 mL/well). The GelMA solutions in the wells were then crosslinked with light (405 nm) (Sovol, Shenzhen, China) at 20 mW cm^−2^ and at a distance of 13 cm from the top of the well for 2 min. The GelMA gels were then flipped, and light crosslinking was repeated on the other side. The crosslinked hydrogels were then cut into approximately 3 mm × 3 mm pieces and frozen at −80 °C or in liquid nitrogen. Once completely frozen, the GelMA pieces were transferred into a pre-chilled mortar and pestle and broken down into finer pieces using a repeated grinding motion. The particles (GelMA MPs) were then collected and lyophilized. The average size of the rehydrated GelMA MPs were obtained by measuring the longest side of irregular-shaped microparticles under an optical light microscope (Echo Revolve, San Diego, CA, USA) and FIJI software (Java 13.0.6, https://imageJ.nih.gov/ij) (NIH).

### 4.2. Culturing Normal Human Articular Chondrocytes

Normal human knee articular chondrocytes (NHACs) were purchased from Lonza Bioscience (Walkersville, MD, USA). NHACs at passages of 2–4 were used for this study. NHACs were cultured in DMEM medium (HyClone, Logan, UT, USA) supplemented with 10% fetal bovine serum (CPS Serum, Kansas City, MO, USA) and 1% 100X antibiotic-antimycotic (Gibco, Gaithersburg, MD, USA). Cells were cultured and passaged in tissue-culture-treated polystyrene plates (TCPs, CELLTREAT, Pepperell, MA, USA) as the standard culture condition.

### 4.3. Preparation of Methacrylated Gelatin Hydrogel Encapsulated with Cells

GelMA hydrogels (GelMA HGs) have been previously used as cell carriers for chondrocytes [24,33,58]. In this study, GelMA HGs were chosen as a comparative cell carrier for other gelatin-based carriers. To encapsulate chondrocytes in GelMA HGs, a previously published procedure was adapted with modifications [58]. Briefly, to prepare 4 GelMA HG samples, 6 μL of 10 mg/mL LAP and 34 μL culture media were added to 80 μL of 15% (*w*/*v*) of GelMA solution containing 36 × 10^4^ chondrocytes. After thorough mixing, 30 μL of this mixture (final GelMA concentration 10%) was transferred to a PDMS mold (5 mm in diameter) and crosslinked for 40 s using a 405 nm light source (Sovol, Shenzhen, China). The gelled GelMA HG disks contained 3 mg of GelMA (dry weight) per disk and 9 × 10^4^ cells/disk. The disks were washed once with PBS and cultured in a 48-well cell-repellent plate (Greiner Bio-One, Monroe, NC, USA) in cell culture medium.

### 4.4. Preparation of Gelatin Microspheres (Gel MSs)

Gelatin microspheres (Gel MSs) were synthesized using a water-in-oil emulsion technique as described previously [36]. In total, 20% of porcine skin gelatin (Type A, Sigma Aldrich, St. Louis, MO, USA) solution was added drop by drop into olive oil (Ward’s Science, Rochester, NY, USA) at 40 °C with constant stirring at 500 rpm. This emulsion was mixed for 30 min and then immediately cooled down at 4 °C. To crosslink the gelatin microspheres, 20 mL of chilled acetone (Sigma Aldrich) combined with 200 μL of 25% glutaraldehyde (Sigma Aldrich) was added slowly using an 18G needle, tubing, and syringe. This solution was chilled at 4 °C for 1 h. The oil and acetone phases were then removed. The fraction of Gel MSs was washed with acetone and centrifuged at 3400 rpm at 4 °C for 5 min. To remove and neutralize the toxic effect of the residual glutaraldehyde, 20 mL of 10 mM glycine was added to the Gel MSs after three washes with acetone [59]. Following glycine treatment, the Gel MSs were washed extensively twice with distilled water. To collect Gel MSs with a size smaller than 100 μm, the washed Gel MS solution was passed through a cell strainer (100 μm, Corning cell strainer, Sigma Aldrich). The passing-through fraction was collected as sorted Gel MSs and lyophilized.

### 4.5. Seeding of Chondrocytes on Different Gelatin-Based Cell Carriers

3 mg of lyophilized Gel MSs or GelMA MPs was sterilized under UV in a biosafety cabinet for 30 min. After UV treatment, samples were transferred into sterile 1.5 mL Eppendorf tubes. A total of 1.0 mL of DMEM complete medium was added to each tube, and tubes were rotated for rehydration at room temperature for 1–2 h. After rehydration, tubes were centrifuged at 5000 rpm, and medium supernatant was removed from each tube. Normal human articular chondrocytes (NHACs) were trypsinized and collected after reaching 80% confluency. A total of 100 μL of 9 × 10^5^/mL NHACs was added to each tube containing cell carriers (9 × 10^4^ cells/sample) or to wells of a 6-well tissue culture plate to serve as the TCP control (9 × 10^4^ cells/well). Cells were mixed with cell carriers using pipetting techniques and incubated in a tissue culture incubator for 30 min with gentle mixing every 10 min. After incubation, 300 μL of complete medium was added to each tube. The mixed cell/carriers were then transferred to wells of a 48-well cell repellent plate (Greiner Bio-One) and cultured statically overnight. After 72 h, the culturing was continued with the plate being tilted in the tissue culture incubator. The media were changed every 3 days.

### 4.6. Pellet Culturing of Chondrocytes in/on Cell Carriers

NHACs were cultured in GelMA HGs or on Gel MSs, GelMA MPs, or TCPs for 7 days. Cells from one well of TCP were trypsinized and transferred to a 5 mL screw cap conical tube (Thermo Fisher Scientific, Somerset, NJ). Cells in or on cell carriers were transferred to conical tubes (one sample per tube). Conical tubes were then centrifuged at 132 g (AllegraTM 6R centrifuge, GF-3.8, Beckman Coulter, Brea, CA, USA) for 5 min to form pellets [20]. In total, 1 mL of complete medium was gently added to each tube. Tubes with a loosen cap were incubated in the tissue culture incubator for 14 days with a change of medium occurring every 3 days.

### 4.7. CalceinAM Staining

To visualize the viable cells with cell carriers or viable cells in pellets, CalceinAM (1 μM in growth medium, Corning Inc., Corning, NY, USA) was added to cells or pellets and incubated for 30 min (45 min for pellet) at 37 °C. After incubation, samples were washed with PBS once and examined under an epi-fluorescence microscope (Echo Revolve, San Diego, CA, USA).

### 4.8. DNA Quantification

After being cultured on different carriers for 7 or 14 days, samples were lysed in 150 µL of Cell Lysis Buffer (Cell Signaling Technology, Danvers, MA, USA) containing 1% SDS. Subsequently, 150 µL of proteinase K digestion solution (50 mM Tris-HCl, pH 8.0, 2 mM CaCl_2_, and 0.4 mg/mL proteinase K (Sigma-Aldrich, St. Louis, MO, USA)) was added to each sample. The samples were then incubated overnight at 55 °C.

To extract DNA, 0.2 mL of PBS was added to each sample, bringing the total volume to 0.5 mL per tube. Next, 0.5 mL of UltraPure™ Phenol:Chloroform:Isoamyl Alcohol (Invitrogen, Carlsbad, CA, USA) was added, followed by thorough mixing and centrifugation at 9660× *g* for 10 min in an IKA G-L centrifuge (IKA Works, Wilmington, NC, USA). The aqueous upper phase was carefully transferred to a fresh tube containing 1 mL of 100% ethanol.

Following overnight incubation at −20 °C, DNA was precipitated by centrifugation at 9660× *g* for 10 min. The resulting DNA pellet was air-dried and dissolved in 50–100 µL of nuclease-free water. Finally, the DNA concentration of each sample was quantified using an AccuClear dsDNA Assay Kit (Biotium, Freemont, CA, USA) following the manufacturer’s protocol.

### 4.9. Alcian Blue Staining and Quantification

To detect the glycosaminoglycan (GAG) formation in pellet cultures after culturing for 14 days, the pellets were stained with Alcian blue 8GX solution (Sigma Aldrich) for 30 min. As staining background controls, Gel MSs or GelMA HGs without cells were also stained. The stained samples were thoroughly washed with PBS to remove any non-bound dye until the wash solution remained clear. Images were captured using an Echo Revolve Microscope (Echo Revolve, San Diego, CA, USA) with a color camera. To quantify the bound Alcian blue dye in pellets, pellets were digested with 0.5 mg/mL of collagenase for 2 h at 37 °C. After digestion, 0.1 mL of sample was transferred to a 96-well plate, and absorbance intensity was measured using a multimode microplate reader (Spark^®^, TECAN, Männedorf, Switzerland) at the wavelength of 600 nm. A standard curve for the Alcian Blue 8GX solution was generated, confirming that the OD readings of all the samples fell within the linear range. The Alcian Blue staining of pellets was quantified as the difference in the optical density (OD) between samples with cells and samples without cells (OD_samples with cells_ − OD_samples without cells_).

### 4.10. Quantitative PCR

The gene expression of chondrocyte markers in cells cultured in/on cell carriers was evaluated using quantitative PCR (qPCR) [20]. NHACs were cultured on TCPs, on Gel MSs, or in GelMA HGs for 14 days. After removing the medium, 0.2 mL of RNA lysis buffer (Promega, Madison, WI, USA) was added to samples. After vigorously mixing using a pipette tip, there were still cells visible in GelMA HGs. To further extract the cells within the hydrogel, GelMA HGs were ground in a mortar with a pestle with liquid nitrogen. RNA isolation, cDNA synthesis, and qPCR analysis were performed using methods described previously [20,52].

### 4.11. Statistical Analysis

For each experiment, 3–4 biological repeats were used, and data were presented as mean ± standard deviation. One-way ANOVA with Tukey’s multiple comparisons test was performed to determine statistical significance using GraphPad Prism version 10.4.1 (accessed on 29 January 2025), GraphPad Software (La Jolla, CA, USA, www.graphpad.com) for all quantitative data. Differences were considered significant at a *p* value of <0.05.

## Data Availability

Data files are included as Supplemental Files.

## Supplementary Materials

The following supporting information can be downloaded at https://www.mdpi.com/article/10.3390/gels11030199/s1: Figure S1: Measurements of microspheres and microparticles. Data files for Figure 5, Figure 6 and Figure 7.
