# Supplementary material for: Expansion and Delivery of Human Chondrocytes on Gelatin-Based Cell Carriers
Source: Gels. 2025 Mar 13;11(3):199. doi: 10.3390/gels11030199 (PMC11942066; doi:10.3390/gels11030199)
Supplement: Supplementary file 1 [file gels-11-00199-s001.zip › Supplemental Figure S1.pdf]

Supplemental Figure S1: Measurement of sizes of Gel MS and GelMA MP

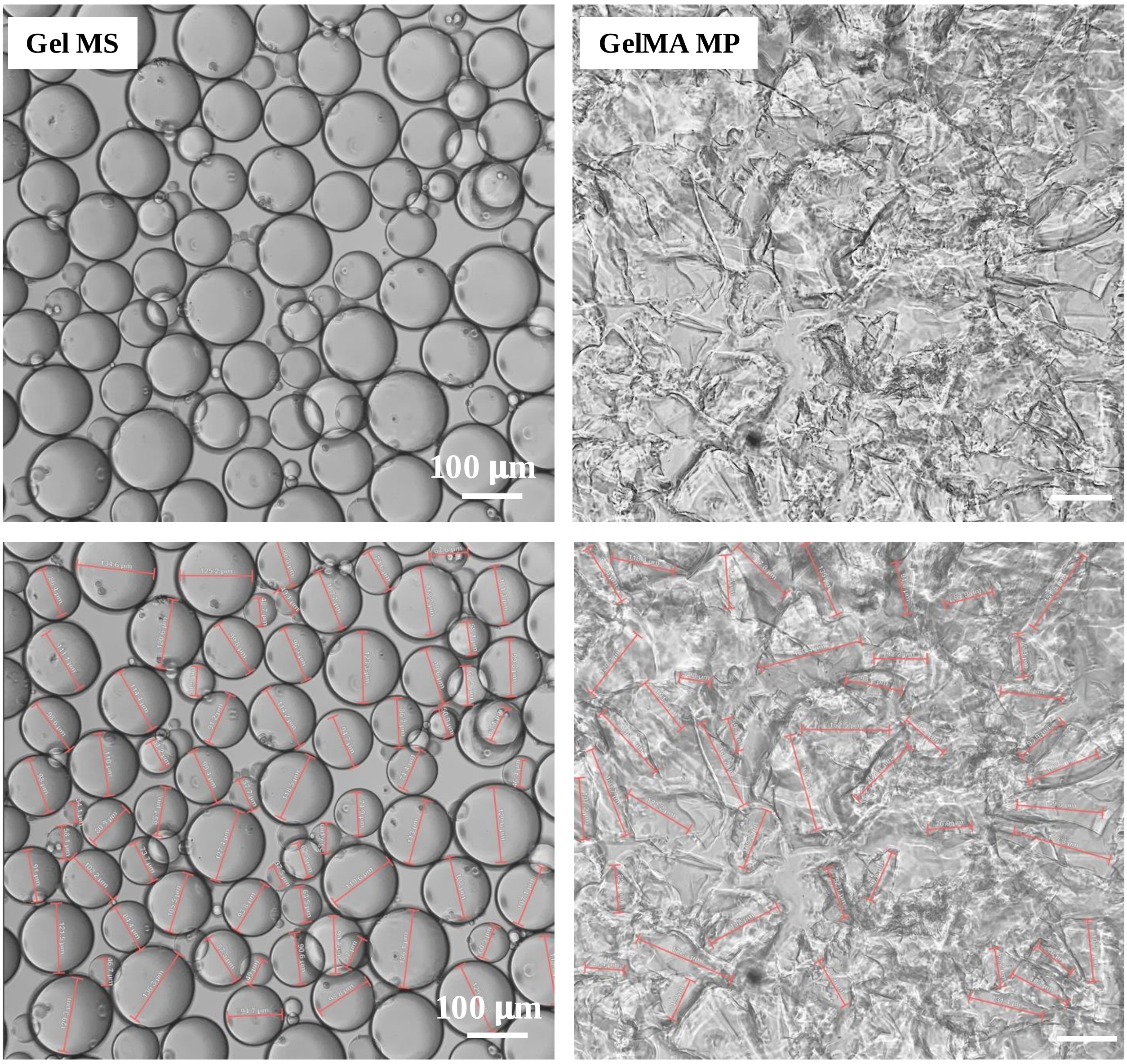

Supplemental Figure S1. Determining the sizes of Gel MS and GelMA MP. Examples images of Gel MS (left panel) and GelMA MP (right panel) were shown. The measurements were captured using the built-in software of ECHO Revolve microscope. Scale bar =100 µm.
